# Increased PHOSPHO1 and alkaline phosphatase expression during the anabolic bone response to intermittent parathyroid hormone delivery

**DOI:** 10.1002/cbf.3772

**Published:** 2022-12-20

**Authors:** Dean A. Houston, Louise A. Stephen, Soher N. Jayash, Katherine Myers, Kirsty Little, Mark Hopkinson, Andrew A. Pitsillides, Vicky E. MacRae, Jose Luis Millan, Katherine A. Staines, Colin Farquharson

**Affiliations:** ^1^ Functional Genetics Division, The Roslin Institute and Royal (Dick) School of Veterinary Studies University of Edinburgh Midlothian UK; ^2^ Comparative Biomedical Sciences The Royal Veterinary College London UK; ^3^ Human Genetics Program Sanford Burnham Prebys Medical Discovery Institute La Jolla California USA; ^4^ School of Applied Sciences, Centre for Stress and Age‐Related Disease University of Brighton Brighton UK

**Keywords:** bone mineralisation, intermittent PTH, phosphatase, PHOSPHO1, SMPD3, TNAP

## Abstract

The administration of intermittent parathyroid hormone (iPTH) is anabolic to the skeleton. Recent studies with cultured osteoblasts have revealed that the expression of PHOSPHO1, *a* bone‐specific phosphatase essential for the initiation of mineralisation, is regulated by PTH. Therefore, this study sought to determine whether the bone anabolic response to iPTH involves modulation of expression of *Phospho1* and of other enzymes critical for bone matrix mineralisation. To mimic iPTH treatment, primary murine osteoblasts were challenged with 50 nM PTH for 6 h in every 48 h period for 8 days (4 cycles), 14 days (7 cycles) and 20 days (10 cycles) in total. The expression of both *Phospho1* and *Smpd3* was almost completely inhibited after 4 cycles, whereas 10 cycles were required to stimulate a similar response in *Alpl* expression. To explore the in vivo role of PHOSPHO1 in PTH‐mediated osteogenesis, the effects of 14‐ and 28‐day iPTH (80 µg/kg/day) administration was assessed in male wild‐type (WT) and *Phospho1^−/−^* mice. The expression of *Phospho1, Alpl, Smpd3, Enpp1, Runx2* and *Trps1* expression was enhanced in the femora of WT mice following iPTH administration but remained unchanged in the femora of *Phospho1^−/−^* mice. After 28 days of iPTH administration, the anabolic response in the femora of WT was greater than that noted in *Phospho1^−/−^* mice. Specifically, cortical and trabecular bone volume/total volume, as well as cortical thickness, were increased in femora of iPTH‐treated WT but not in iPTH‐treated *Phospho1^−/−^* mice. Trabecular bone osteoblast number was also increased in iPTH‐treated WT mice but not in iPTH‐treated *Phospho1^−/−^* mice. The increased levels of *Phospho1, Alpl, Enpp1* and *Smpd3* in WT mice in response to iPTH administration is consistent with their contribution to the potent anabolic properties of iPTH in bone. Furthermore, as the anabolic response to iPTH was attenuated in mice deficient in PHOSPHO1, this suggests that the osteoanabolic effects of iPTH are at least partly mediated via bone mineralisation processes.

## Significance statement

Intermittent administration of parathyroid hormone (iPTH) is recognised to increase osteoblast number and promote bone formation. A critical component of this anabolic response is the mineralisation of the osteoid matrix but the effects iPTH has on the expression of mineralisation regulating enzymes is unclear. This study's principal finding was that iPTH administration increased the expression of *Phospho1, Alpl, Enpp1* and *Smpd3* in vivo which was consistent with a bone anabolic PTH regimen. The data also disclosed that in the absence of PHOSPHO1, the iPTH anabolic response was dampened, suggesting that amplified *Phospho1* expression is a prerequisite for a full iPTH‐mediated bone anabolic response.

## 1 INTRODUCTION

Parathyroid hormone (PTH) is synthesised and secreted by the chief cells of the parathyroid glands to maintain serum calcium (Ca2+) concentrations within a very narrow range. This function is essential to prevent disturbance to a range of cellular functions and is mediated by interactions with the PTH1 receptor in key target tissues such as bone, kidney and intestine.^1^, ^2^ PTH also maintains phosphate homoeostasis by several mechanisms including the promotion of osteocyte synthesis of ﬁbroblast growth factor 23 (FGF23) which, in conjunction with cofactor klotho and via the renal FGF receptor, promotes phosphaturia by downregulating sodium–phosphate cotransporter (Npt2a and Npt2c) expression.^3^

In addition to increasing osteoclast bone resorption to mobilise Ca2+, PTH also has profound effects on the cells of the osteoblast lineage and is now regarded as an important hormone regulating bone remodelling.^4^ The pleiotropic effects of PTH on bone are dictated by the frequency of the exposure, and the mechanisms involved have been progressively uncovered, aiding our understanding of the osteo‐anabolic effects of intermittent administration of PTH (iPTH). The pioneering studies by Reeve et al.^5^ revealed increased bone formation in osteoporotic women in response to daily exposure to low‐dose PTH 1–34 (100 µg/day human PTH). Extensive clinical trials have also confirmed the efficacy of daily PTH (1–34) injection in reducing vertebral and nonvertebral fractures and increasing bone mineral density (BMD) in postmenopausal osteoporotic women.^6^, ^7^, ^8^ In contrast, continuous delivery results in increased bone loss and increased porosity principally in the cortical compartment.^9^, ^10^, ^11^ This bone loss is associated with increased bone remodelling where the enhanced expression of receptor activator of nuclear factor‐ƙB ligand (RANKL) expression and the decreased expression of the RANKL decoy receptor, osteoprotegerin, ensure that any increases in bone formation are dwarfed by a prevailing bone resorption response.^12^, ^13^

The capacity of continuous or iPTH delivery to finely tune osteoblast and osteocyte gene expression is likely to contribute to the pleiotropic effects of PTH on the skeleton.^10^, ^14^ The osteoblast and its progenitors have reproducibly been shown to be the primary in vivo target for PTH. iPTH administration promotes osteoblastogenesis and the treatment of cultured bone marrow cells and calvarial osteoblasts by iPTH results in increased expression of Runt‐related transcription factor 2 (*Runx2*), type I collagen procollagen (*Col1a1*), osteocalcin (*Bglap*) and tissue‐nonspecific alkaline phosphatase (*Alpl*).^10^, ^15^, ^16^ iPTH administration also reduces osteoblast apoptosis, reactivates quiescent bone‐lining cells and downregulates sclerostin expression.^17^, ^18^

Whilst the effects of PTH on the expression of osteoblast transcription and differentiation factors have been studied widely, less is known about the impact of PTH on the expression of genes that encode mineralisation‐regulating enzymes. Such knowledge would help clarify the function of PTH on the mineralisation process per se rather than osteoblast differentiation and matrix production during bone formation. Although hyperparathyroidism is associated with increased bone remodelling and decreased BMD, the bone formation response that occurs is predominated by osteoid production rather than the formation of a true mineralised bone matrix.^19^ Furthermore, rats receiving an anabolic PTH regimen for 3 weeks have increased osteoid surface, thickness and volume which is consistent with an increased rate of bone formation.^20^

The initiation and propagation of the mineral phase within newly formed osteoid is dependent upon the interplay between PHOSPHO1 and TNAP which are required for the generation of Pi within matrix vesicles (MVs) and the hydrolysis of the mineralisation inhibitor, PPi, respectively.^21^, ^22^, ^23^ nSMase2 catalyses the hydrolysis of sphingomyelin within MV membranes to produce phosphocholine; a recognised substrate for PHOSPHO1^24^, ^25^ whereas ENPP1 generates PP_i_ from nucleoside triphosphates.^26^ PTH alters the gene and protein expression of *Phospho1*, *Smpd3* (encoding the protein nSMase2), *Alpl* and *Enpp1*. Specifically, *Phospho1* and *Smpd3* expression levels are decreased whereas *Alpl* expression is increased in MC3T3‐C14 osteoblast‐like cells after 24 h of continuous PTH exposure whereas *Enpp1* expression by differentiated IDG‐SW3 osteocyte‐like cells was decreased after 3–6 h of PTH treatment.^27^, ^28^ These data confirm previous studies reporting the decreased expression of *Phospho1, Smpd3* and other mineralisation‐associated genes by PTH (up to 24 h exposure) in both osteocyte and bone marrow stromal cell lines.^29^, ^30^

Whilst the downregulation of *Phospho1* and *Smpd3* in osteoblasts in response to continuous PTH may provide mechanistic insight for the observed lower BMD in hyperparathyroidism, these studies are limited by the use of cell lines and do not provide insight into the anabolic effects of iPTH on the expression of mineralisation regulating enzymes. This study, therefore, sought to determine whether iPTH alters the expression of *Phospho1*, *Smpd3* and *Alpl* in primary osteoblasts and murine bones. We also completed studies in *Phospho1* null mice to help resolve the role and regulation of PHOSPHO1 in the iPTH anabolic regimen.

## 2 METHODS

### 2.1 Animal studies

Phospho1‐R74X‐null mutant mice (*Phospho1* KO) were generated and maintained as previously described.^31^ This strain carries a C>T mutation in codon 74 of exon 2 of the *Phospho1* gene leading to a premature stop codon (R74X) and the complete absence of PHOSPHO1 protein.^31^ All animal experiments were approved by the Roslin Institute's named veterinary surgeon and named animal care and welfare officer (NACWO), with animals maintained in accordance with the Home Office code of practice (for the housing and care of animals bred, supplied, ARRIVE guidelines or used for scientific purposes). We chose to study male mice during juvenile development as male mice show a greater response to iPTH than female mice and skeletal abnormalities are present within *Phospho1* KO mice of this age.^31^, ^32^, ^33^ Indeed, the observed age‐related defects in trabecular architecture and compromised cortical microarchitecture observed in bones from *Phospho1* KO mice are rectified by skeletal maturation.^34^

### 2.2 PTH administration

#### 2.2.1 Six hours PTH

Twenty‐eight‐day‐old male C57BL/6 wild‐type (WT) mice received a single subcutaneous injection of bovine PTH (1–34) (Sigma; 80 µg/kg) (*n* = 4), or vehicle control (99.8% physiological saline, 0.2% bovine serum albumin) (*n* = 4), and culled 6 h later. The left femur was dissected, epiphyses removed and marrow eliminated by centrifugation before snap freezing in liquid nitrogen and storage at −80°C for subsequent gene expression analysis. The right tibia was fixed in paraformaldehyde (PFA) for histomorphometry analysis.

#### 2.2.2 iPTH

Twenty‐eight‐day‐old male WT and *Phospho1* KO mice received a subcutaneous injection of either 80 µg/kg bovine PTH (1–34) or vehicle control once daily for 14 or 28 (*n* = 4–5) days. Twenty‐four hours after the final PTH injection, mice were culled and the left femur was dissected, epiphyses removed and marrow eliminated before snap freezing in LN_2_ and storage of the diaphysis at −80°C for RNA extraction. The left tibia was dissected and stored in dH_2_0 at −20°C for microcomputed tomography (μCT) analysis. The right tibia was fixed in PFA for histomorphometry analysis.

### 2.3 μCT

Scanning of mice tibiae was performed with an 1172 X‐Ray Microtomograph (Skyscan) to evaluate cortical and epiphyseal trabecular bone geometry. High‐resolution scans with an isotropic voxel size of 5 µm were acquired (55 kV, 0.5 mm aluminium filter, 0.6° rotation angle, 2 frame averaging). The scans were reconstructed using NRecon software (Skyscan) and a median filter applied to the images before reconstruction to remove artefacts, including beam‐hardening and ring artefacts. Data were analysed with CtAn software (Skyscan). The threshold values for the µCT analysis of trabecular bone were chosen to be between 60 and 255 and between 100 and 255 for cortical bone. The calibrated values upon which the greyscale range was set on were 0–2.34 g/cm^3^. Metaphyseal trabecular bone of the proximal tibia was assessed in a 1000 µm section, 5% of the total bone length below the first appearance of a trabecular ‘bridge’ connecting the two primary spongiosa bone islands.^34^ Cortical bone was assessed in a 500 µm section at 37% of the total bone length from the reference starting slice (first appearance of the medial tibial condyles). To assess BMD, BMD phantoms were used to calibrate the CTAn software. BMD phantoms of known calcium hydroxyapatite mineral densities of 0.25 and 0.75 g/cm^3^ were scanned and reconstructed using the same parameters as used for bone samples.

### 2.4 Histomorphometry

Tibiae were fixed overnight in 4% PFA (pH 7.2) and decalcified in 10% EDTA (pH 7.4) for 14 days at 4°C with constant agitation. Samples were processed to paraffin wax using a Leica ASP300S tissue processor (Leica Microsystems). Wax blocks were sectioned using a Leica RM2235 microtome equipped with MX35 Premier plus microtome blades (Thermo Fisher Scientific). Five micrometres wax sections were stained by haematoxylin and eosin (H&E) and imaged using a NanoZoomer‐XR slide scanning system (Hamamatsu Photonics). Osteoblasts on trabecular bone surfaces (N.Ob/BS [1/mm]) were quantified in a 1 mm section starting 150 µm below the growth plate, using the BIOQUANT OSTEO (BIOQUANT Image Analysis Corporation) software package.

### 2.5 Calvarial osteoblast isolation and cell culture

Osteoblasts were isolated from calvaria of 3–5 days old WT mice following established procedures.^35^, ^36^ In brief, excised calvaria were digested with 1 mg/ml collagenase type II for 10 min followed by 1 mg/ml collagenase (30 min), 4 mM EDTA (10 min) and 1 mg/ml collagenase (30 min). The first digest was discarded and the cells from the other digests were pooled and plated and expanded in αMEM containing 10% foetal bovine serum, and 0.5% gentamicin (Invitrogen). Once semiconfluent (~80%), the osteoblasts were trypsinised and plated in six‐well plates (Thermo Fisher Scientific) at 1 × 10^4^/cm². When confluent, the culture media was supplemented with 50 µg/ml ascorbic acid and 2.5 mM β‐glycerophosphate to induce differentiation and support matrix mineralisation. Cell culture medium was replaced every 2–3 days for the duration of the experiments. Bovine PTH (1–34) (50 nM) was added to the cultures on Days 4, 11 and 18 for 24 h before each of the time points investigated. This concentration of PTH was based on our previous studies where 50 nM PTH had the maximum effect on the gene expression of MC3T3 osteoblast‐like cells.^27^ In further experiments, primary osteoblasts were treated with 50 nM PTH for 6 h in every 48 h period for 8 days (4 cycles), 14 days (7 cycles) and 20 days (10 cycles) in total.^10^, ^16^ Cultured cells were scraped from individual plates into 1 ml of ice‐cold phosphate‐buffered saline, pelleted and stored at −80°C.

### 2.6 RNA extraction and RT‐qPCR analysis

Total RNA was extracted from cells or whole bones using the RNeasy mini kit (Qiagen) according to the manufacturer's instructions. RNA concentration was assessed by absorbance at 260 nm and purity by A260/280 ratio using a NanoDrop spectrophotometer (ND‐1000, Thermo Fisher Scientific). The reverse transcription of RNA to cDNA was accomplished using Superscript II (Invitrogen) according to the manufacturer's instructions. RT‐qPCR reactions, using the SYBR green detection, were completed in a 96‐well PCR plate and cycled in a Stratagene Mx3000P real‐time qPCR system (Stratagene). Samples were assessed in triplicate and normalised against *Atp5b* as previously reported using murine calvarial osteoblasts.^27^, ^32^ The relative expression of the target genes was calculated using the ΔΔCt method and expressed as a fold change compared to control.^37^ The primer pairs used to amplify *Atp5b, Phospho1, Smpd3, Alpl, Runx2, Enpp1, Sost, Trps1* and *PthR1* are listed in Table 1.

**Table 1:** qPCR primers used for gene expression studies

Gene	Source		Sequence (5′−3′)
*Phospho1*	Primer Design	F	TTCTCATTTCGGATGCCAACA
		R	TGAGGATGCGGCGGAATAA
*Smpd3*	MWG Eurofins	F	CTAACCACCCACTCCCACTT
		R	CCAATAGACTCCAAACCTGAAGA
*Alpl*	MWG Eurofins	F	GGGACGAATCTCAGGGTACA
		R	AGTAACTGGGGTCTCTCTCTTT
*Pthr1*	MWG Eurofins	F	CCAACCCTAAGCATCCCAAA
		R	TCCTCGGAGACTGGTAATGG
*Runx2*	MWG Eurofins	F	TTCTTCACACGCATTCCATCT
		R	GCCAACAGTAAAGTCACAATCC
*Sost*	MWG Eurofins	F	ATACCACAATACTGAATCTGAAAGC
		R	CACTATTTGCCTGTCCCTCTG
*Trps1*	MWG Eurofins	F	GAGCCCAATCACGTTTCAGTT
		R	TCCTTCCTGCTTCTTGCTAGC
*Enpp1*	MWG Eurofins	F	GCTAATCATCAGGAGGTCAAG
		R	GCTAATCATCAGGAGGTCAAG
*Atf5b*	Primer Design	F	Not disclosed
		R	Not disclosed

### 2.7 Statistical analysis

Data are presented as mean ± SEM of at least 3–4 replicate wells per in vitro experiment and 4–5 mice per in vivo study. Graphpad prism 6 was used to carry out the statistical analysis of the data. Direct comparison between two sets of data was made by Student's *t*‐test. For multiple comparisons, data were analysed for statistical significance by two‐way analysis of variance with Bonferroni post hoc correction.

## 3 RESULTS

### 3.1 iPTH treatment decreases Phospho1, Alpl and Smpd3 expression in primary osteoblasts

**Figure 1:**
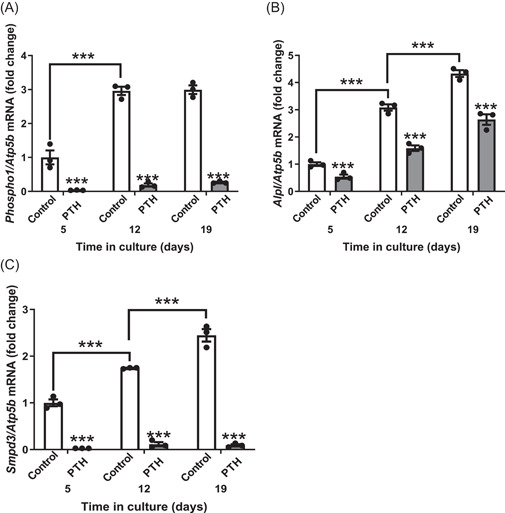
The effect of iPTH on the expression of mineralisation regulating enzymes during osteoblast matrix mineralisation. (A) *Phospho1* (B) *Alpl* (C) *Smpd3* expression in response to 24 h of 50 nM PTH exposure by primary osteoblast cells at different stages of differentiation (Days 5, 12, 19 postconfluency). Data are represented as mean ± SEM, *n* = 3/treatment/time point; ***p < .001. iPTH, intermittent parathyroid hormone.

**Figure 2:**
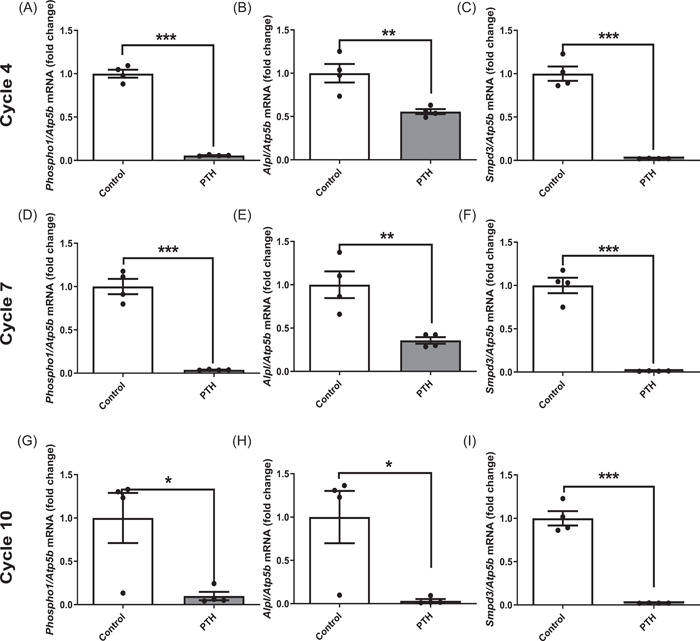
The effects of iPTH on the expression of mineralisation regulating enzymes during osteoblast matrix mineralisation. PTH (50 nM) was administered to primary osteoblasts in cycles (6 h PTH treatment in every 48 h period for up to 20 days). For cycle 4: (A) *Phospho1* (B) *Alpl* (C) *Smpd3*; cycle 7 (D) *Phospho1* (E) *Alpl* (F) *Smpd3*; cycle 10 (G) *Phospho1* (H) *Alp1* (I) *Smpd3*. Data are represented as mean ± SEM, *n* = 4/treatment/time point; *p < .05; **p < .01; ***p < .001. iPTH, intermittent parathyroid hormone.

In accordance with previous studies using MC3T3 cells, the expression of *Phospho1, Alpl* and *Smpd3* by primary osteoblasts increased with time in culture (*p* < .001; Figure 1).^27^ However, the expression of all three genes was significantly decreased (*p* < .001) by 24 h PTH treatment at all stages of osteoblast maturation studied (Figure 1A–C). To better mimic iPTH treatment, we next treated primary osteoblasts to a number of iPTH treatment cycles. *Phospho1, Alpl* and *Smpd3* expression were all decreased at Day 8 (4 iPTH cycles), Day 14 (7 iPTH cycles) and Day 20 (10 iPTH cycles) (Figure 2). The expression of both *Phospho1* and *Smpd3* was almost completely inhibited after 4 iPTH cycles and this was maintained for the duration of the study. In contrast, 10 iPTH cycles was required to get a similar response for *Alpl* expression (Figure 2). These in vitro data indicate that PTH delivered via a single or repeated iPTH challenge results in decreased expression of *Phospho1, Alpl* and *Smpd3* by primary osteoblasts.

### 3.2 Single PTH delivery to WT mice induces a rapid increase in osteoblast Phospho1, Alpl and Smpd3 expression

**Figure 3:**
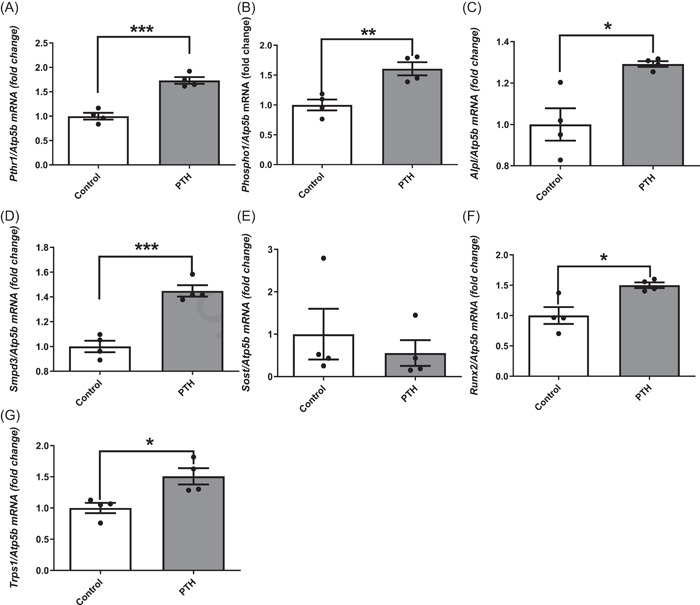
The effects of a single dose of PTH on gene expression within the femora of WT mice. (A) *Pth1r* (B) *Phospho1* (C) *Alpl* (D) *Smpd3* (E) *Sost* (F) R*unx2* and (G) *Trps1* expression after 6 h following a single dose of PTH (80 µg/kg). Data are represented as mean ± SEM (*n* = 4). *p < .05; **p < .01; ***p < .001 compared to control. PTH, parathyroid hormone; WT, wild‐type.

**Figure 4:**
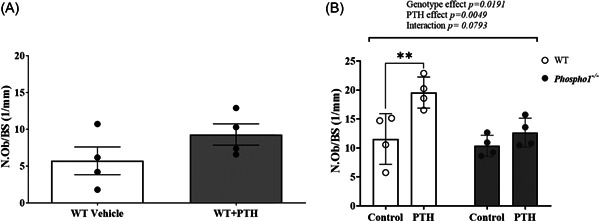
The effects of iPTH on trabecular bone osteoblast number within the tibia of WT and *Phospho1* ^−/−^ mice. (A) after 6 h following a single dose of PTH (80 µg/kg) (B) after 28 days of intermittent PTH (80 µg/kg) or vehicle control. Data are represented as mean ± SEM (*n* = 4). **p < .01 compared to control. iPTH, intermittent parathyroid hormone; WT, wild‐type.

To determine whether the rapid inhibitory effects of PTH on *Phospho1, Alpl* and *Smpd3* expression noted in isolated primary osteoblasts translated to the in vivo situation, we investigated gene expression within the femora of 4‐week‐old, male WT mice after a 6 h exposure to a single subcutaneous injection of PTH (80 µg/kg). This dosing regimen resulted in a rapid increase in *Pthr1* (*p* < .001; Figure 3A) and *Phospho1* (*p* < .01; Figure 3B) expression compared with vehicle‐treated animals. The expression of *Alpl* (*p* < .05; Figure 3C) and *Smpd3* (*p* < .001; Figure 3D) were likewise significantly increased in response to PTH treatment. *Sost* expression displayed a trend towards inhibition by PTH exposure, but this did not reach statistical significance (Figure 3E). The increased expression of the osteoblast transcription factors, *Runx2* (*p* < .05; Figure 3F) and *Trps1* (*p* < .05; Figure 3G) in response to a single PTH dose is consistent with its bone anabolic actions. Osteoblast number on the trabecular bone surface was similar between the control and PTH‐administered mice (Figure 4A).

### 3.3 Fourteen‐day iPTH administration increases Phospho1, Alpl and Smpd3 expression in WT mice only

Having shown that a single delivery of PTH rapidly increases *Phospho1, Alpl and Smpd3* expression within femora, we next determined if the anabolic bone response typical of an iPTH regimen was also associated with changes to the expression of these key regulators of bone mineralisation. Also, as PHOSPHO1 is recognised to be essential for the initiation of the mineralisation process, we also aimed to establish if amplified *Phospho1* expression in response to iPTH was a prerequisite for a bone anabolic response by examining whether the genetic ablation of PHOSPHO1 attenuated the iPTH anabolic response.

**Figure 5:**
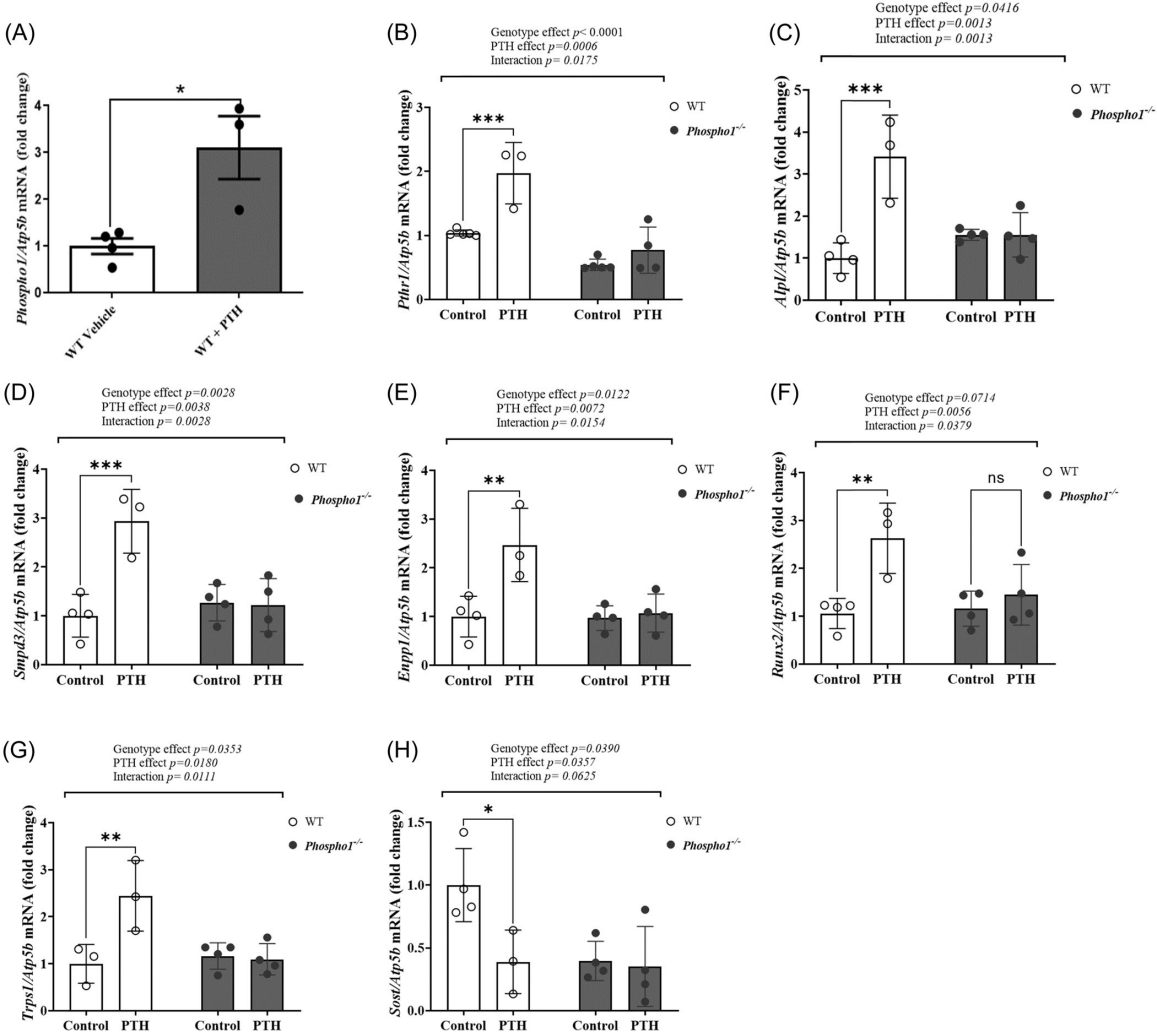
The differential effects of 14‐days iPTH on gene expression within the femora of WT and *Phospho1^−/−^* mice. Gene expression analysis within the femora of WT and *Phospho1^−/−^* mice receiving iPTH (80 µg/kg/day) for 14 days. (A) *Phospho1* (B) *Pth1r* (C) *Alpl* (D) *Smpd3* (E) *Enpp1* (F) *Runx2* (G) *Trps1* and (H) *Sost*. Data are represented as mean ± SEM (*n* = 3–4). *p < .05; **p < .01; ***p < .001. iPTH, intermittent parathyroid hormone; WT, wild‐type.

In the diaphysis of WT animals, iPTH administration for 14 days induced a 3.1‐fold increase in *Phospho1* expression compared to vehicle‐treated control animals (*p* < .05; Figure 5A). Similarly, *Pthr1* (2.0‐fold; *p* < .001; Figure 5B), *Alpl* (3.4‐fold; *p* < .001; Figure 5C), *Smpd3* (2.9‐fold; *p* < .001; Figure 5D) and *Enpp1* (2.4‐fold; *p* < .01; Figure5E) expression were all increased in WT mice by iPTH compared to vehicle‐treated control mice. In contrast, the diaphysis from iPTH and vehicle‐treated *Phospho1* KO mice both had similar expression levels of *Pthr1*, *Alpl, Smpd3* and *Enpp1* resulting in the PTH effect being significantly modified by *Phospho1* deficiency (Figure 5B–E). *Runx2* (2.6‐fold; *p* < .01; Figure 5F) and *Trps1* (2.4‐fold; *p* < .01; Figure 5G) expression was increased in response to iPTH administration in WT but not *Phospho1* KO mice resulting in a significant interaction between PTH treatment and genotype (Figure 5F,G). As expected, WT mice receiving iPTH expressed less *Sost* compared to vehicle‐treated WT mice (*p* < .05; Figure 5H). Furthermore, the basal expression of *Sost* mRNA in *Phospho1* KO mice was significantly reduced compared to WT mice but there was no further decrease in *Sost expression* in response to iPTH administration (*p* < .05; Figure 5H). When the data are compared to the results of the single 6 h PTH in vivo study (Figure 3), the effects of iPTH on gene expression in WT mice were all accentuated with 14 days iPTH treatment (Figure 5).

To determine if the changes in gene expression were reflected in a bone anabolic response, the effects of 14‐day iPTH exposure on bone microarchitecture, geometry and BMD within both WT and *Phospho1* KO tibiae was determined (Table 2). Increased trabecular thickness and BMD and decreased trabecular pattern factor were noted in both WT and *Phospho1* KO mice in response to iPTH but no other changes in the trabecular compartment were noted. The anabolic effects of iPTH on cortical bone of mice treated for 14 days were also limited although there was a clear genotype effect on many of the measured parameters (Table 2).

**Table 2:** Micro‐CT analysis from bones of wild‐type and *Phospho1^−/−^* mice treated with vehicle and iPTH for 14 days

	WT		WT + iPTH		Phospho1^−/−^		Phospho1^−/−^ + iPTH		*p* Value		
	Mean	SEM	Mean	SEM	Mean	SEM	Mean	SEM	Interaction	PTH effect	Genotype effect
Tb. BV/TV (%)	2.94	0.42	4.28	0.41	2.95	0.30	3.67	0.38	.4335	.0196	.4484
Tb. Th (µm)	43.86	0.75	48.71^a^	0.44	39.88	1.33	45.92^b^	1.35	.5993	.0003	.0097
Tb. Sp. (mm)	0.49	0.04	0.55	0.07	0.43	0.02	0.44	0.074	.7035	.5623	.1388
Tb. N (mm^−1^)	0.79	0.12	0.70	0.14	0.87	0.02	1.05	0.21	.4472	.5949	.1891
Tb. Pf. (mm^−1^)	31.93	1.97	25.86^a^	0.76	31.67	1.06	23.81^b^	1.31	.5468	.0005	.4381
Tb. BMD (g/cm^3^)	0.28	0.01	0.33^a^	0.01	0.24	0.01	0.30^b^	0.02	.5662	.0002	.0086
Ct. BV/TV (%)	49.94	0.26	50.32	3.80	53.41	3.02	53.66	3.96	.9849	.9246	.3231
Ct. Th (µm)	127.9	7.37	171.3^a^	10.24	111.3	14.02	108.7	8.78	.0529	.0804	.0033
Ct. Ar (mm^2^)	0.76	0.05	0.76	0.03	0.60	0.03	0.73^b^	0.02	.1074	.1142	.0194
Tt. Ar (mm^2^)	1.53	0.11	1.53	0.06	1.14	0.09	1.37	0.08	.2079	.2253	.0105
Ma. Ar (mm^2^)	0.77	0.05	0.76	0.09	0.54	0.08	0.64	0.09	.4842	.5111	.0426
Ct. BMD (g/cm^3^)	2.62	0.02	2.63	0.04	2.55	0.02	2.57	0.01	.3143	.0819	.0021

Abbreviations: BMD, bone mineral density; BV/TV, bone volume/total volume; CT, computed tomography; iPTH, intermittent parathyroid hormone; WT, wild‐type.

^a^ *p* < .05 from WT.

^b^ *p* < .05 from Phospho1^−/−^ KO.

### 3.4 Loss of PHOSPHO1 blunts the bone anabolic response to iPTH

**Figure 6:**
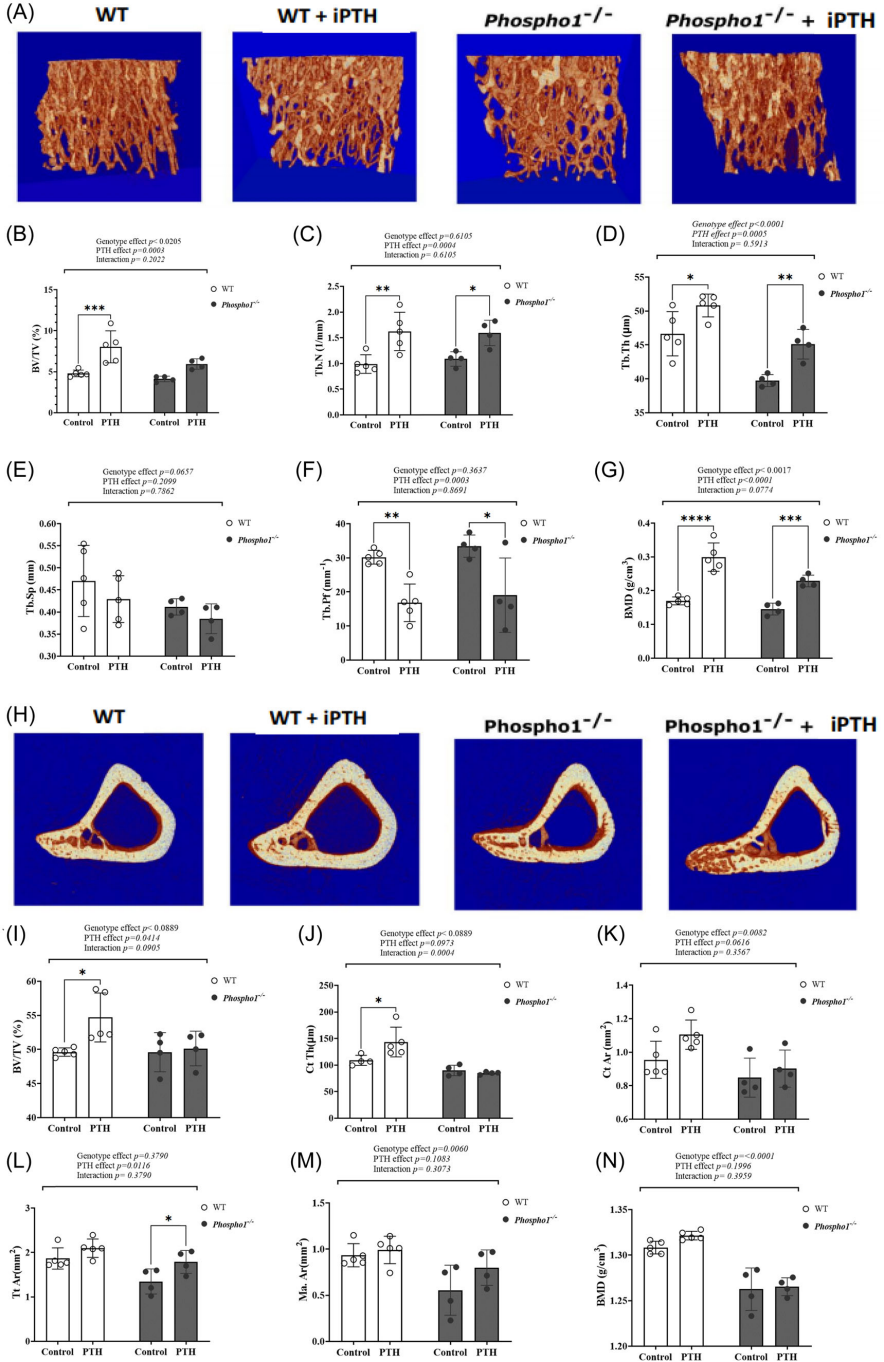
Trabecular and cortical bone parameters in WT and *Phospho1^−/−^* mice receiving iPTH or vehicle control for 28 days. Analysis of the trabecular bone parameters (A–G); (B) trabecular bone volume/total volume (BV/TV) (C) trabecular number (Tb.N.) (D) trabecular thickness (Tb.Th.) (E) trabecular separation (Tb.Sp.) (F) trabecular pattern factor (Tb.Pf) (G) trabecular bone mineral density (BMD). Analysis of the cortical bone parameters (H–N); (I) cortical bone volume/total volume (BV/TV) (J) cortical thickness (Ct.Th) (K) cortical area (Ct.Ar) (L) total cortical tissue area (Tt.Ar) (M) medullary area (Ma.Ar) (N) cortical BMD. Data are represented as mean ± SEM (*n* = 4–5). *p < .05; **p < .01; ***p < .001; ****p < .0001. iPTH, intermittent parathyroid hormone; WT, wild‐type.

Although daily injections of PTH for 14 days results in increased expression of *Phospho1*, *Smpd3* and *Alpl* in femora, there was little evidence of anabolic bone changes. It is likely that whilst 14 days iPTH was sufficient to observe changes in gene expression, anabolic changes to the skeleton are not induced within this timescale. Therefore, we next administered iPTH to WT and *Phospho1* KO mice for 28 days to determine if there was a modified response to iPTH administration in the absence of PHOSPHO1. The percentage trabecular bone volume (BV/TV) (*p* < .001; Figure 6B) was increased in iPTH‐treated WT mice but not in iPTH‐treated *Phospho1* KO mice but there was no significant overall interaction between PTH and genotype. Trabecular number, thickness and BMD were increased whereas trabecular pattern factor was decreased by iPTH in WT mice and these responses were similarly observed in *Phospho1* KO mice (Figure 6C,D,F,G). Trabecular separation in WT and *Phospho1* KO mice was not affected by iPTH (Figure 6D). After 28 daily injections of PTH, the anabolic response in cortical parameters was now evident in WT mice. Cortical BV/TV (*p* < .05; Figure 6I) and cortical thickness (*p* < .05; Figure 6J) were increased in iPTH‐treated WT but not in iPTH‐treated *Phospho1* KO mice and for cortical thickness there was a significant interaction between PTH treatment and genotype. Total cortical tissue area was increased only in iPTH‐treated *Phospho1* KO mice (Figure 6L; *p* < .05). Cortical area, medullary area and BMD in both WT and *Phospho1* KO mice were not affected by iPTH but there was a significant genotype effect on all three parameters (Figure 6K,M,N). Osteoblast number on the trabecular bone surface was increased (*p* < .05) by iPTH in WT but not *Phospho1* KO mice but there was no significant overall interaction between PTH and genotype (Figure 4B). These findings show that the anabolic bone response to iPTH is linked to changes in the expression of these key regulators of mineralisation and are attenuated by the genetic ablation of PHOSPHO1.

## 4 DISCUSSION

Cells of the osteoblast lineage are responsible for the osteo‐anabolic effects of iPTH. iPTH reduces osteoblast apoptosis and promotes the commitment and differentiation of mesenchymal cells and osteoblast precursors to progress through the osteoblast lineage.^38^ Lineage tracing experiments confirmed that iPTH exposure induces the reactivation of bone‐lining cells to functional osteoblasts and delays the transition of mature osteoblasts to bone‐lining cells.^18^ Osteocytes are also targets of PTH where it inhibits sclerostin expression to promote bone formation through the WNT/β‐catenin signalling pathway.^39^, ^40^ In addition to these established pathways by which iPTH promotes BMD it is conceivable that iPTH may directly alter the expression levels of genes that impact on the mineralisation process.^27^, ^28^, ^29^, ^30^ The deletion of *Smpd3*,^24^, ^41^ *Phospho1* ^32^, ^42^, ^43^ or *Alpl* ^44^, ^45^ results in a hypomineralised skeleton, whereas in mice with an ablation of both *Phospho1* and *Alpl* there is a complete absence of a mineralised skeleton.^31^ The aims of this study were, therefore, to fully assess the effects of iPTH delivered in vitro and in vivo on the expression of *Phospho1, Alpl* and *Smpd3* to better understand the response of osteoblasts to an anabolic PTH regimen. The expression of *Phospho1, Alpl* and *Smpd3* expression in the femur were all enhanced in response to either a single (6 h) or daily (14 days) regimen of PTH administration in WT mice. These gene changes in response to iPTH in vivo are consistent with the anabolic bone response that was most notable after 28 days of iPTH although trabecular BMD was increased after 14 and 28 days of daily PTH administration. Similar responses in trabecular bone architecture and BMD in response to 21 days of iPTH treatment have been reported previously in mice.^20^ These in vivo gene expression results are in contrast to those observed in vitro where the expression of *Phospho1, Alpl* and *Smpd3* in primary calvarial osteoblasts were all found to be downregulated by iPTH treatment. There are a number of reasons to possibly explain the disparity between the in vitro and in vivo data. Firstly, PTH is cleared very rapidly in vivo, and therefore it is possible that a 6 h iPTH treatment in vitro may induce a more continuous‐like PTH response; a treatment known to decrease both TNAP and PHOSPHO1 expression in cultured murine primary osteoblasts.^46^ Furthermore, iPTH promotes T‐cells to secrete WNT10b which has a major role in the regulation of osteoblast number and bone mass. This regulatory pathway cannot be recapitulated in osteoblast cultures as used in this study.^47^ A decrease in *Phospho1* and *Smpd3* expression by osteoblast‐ (MC3T3‐C14) and osteocyte‐ (IDG‐SW3 and Kusa4b10) like cells has previously been noted in response to PTH exposure for 6 or 24 h providing confidence that our in vitro data is robust despite the lack of biological repeats.^27^, ^29^, ^30^ However, our data do not necessarily align with other studies where a similar iPTH protocol caused an increase in *Alpl* expression and TNAP activity as well as an increased number of mineralised nodules.^10^, ^16^ These discrepancies are difficult to reconcile but the decrease in the expression of these genes in cultured cells may reflect their direct downregulation by PTH, whereas the raised *Phospho1, Smpd3* and *Alpl* expression levels after 14 days of daily PTH administration in vivo may reflect the increased abundance of newly formed osteoblasts actively producing and mineralising their matrix. However, the direct regulation of these genes by PTH cannot be ruled out as an increase in the expression of *Phospho1, Smpd3* and *Alpl* were also noted in the femur within 6 h of PTH treatment in the absence of increased osteoblast numbers. Rapid changes in the expression of *Phospho1* and *Smpd3* have also been reported in the tibia of rats administered PTH for 1.5, 4 and 6.5 h.^20^

Despite the lack of change in osteoblast numbers after 6 h of PTH treatment, the expression of both *Runx2* and *Trps1* was upregulated, with both known to regulate *Smpd3* and *Phospho1* expression in osteoblasts.^48^, ^49^ *Trps1*, a GATA transcription factor which primarily acts to repress genes known to be associated with the bone mineralisation process.^48^, ^50^ Mutations in the *Trps1* gene lead to the human condition tricho‐rhino‐phalangeal syndrome which is characterised by craniofacial and skeletal dysplasias and siRNA mediated knockdown of *Trps1* in a pre‐odontoblastic cell line leads to the suppression of *Phospho1, Alpl* and *Smpd3* expression.^48^ Also, *Runx2*, an osteoblast transcription factor upstream of *osterix* expression, is essential for osteoblastogenesis and its overexpression in mouse limb bud cultures leads to a >3‐fold increase in *Phospho1* and *Smpd3* expression.^49^ Therefore, higher osteoblast *Trps1* and *Runx2* expression may explain the rapid increase in *Phospho1* and *Smpd3* expression in bones of PTH‐treated mice which do not show evidence of increased osteoblast numbers.

The data from this study also reveals that the anabolic response to iPTH was blunted in mice deficient in PHOSPHO1. The reasons for this are unclear and are likely to be complex but of interest, a number of other gene deletions such as *Prg4, Alpl, Lrp5* and *Lrp6* have also been shown to attenuate the iPTH anabolic response.^33^, ^51^ To confirm the anabolic response, future studies should include histomorphometry and serum analyses of systemic markers including bone turnover markers. It should be noted that some significant interactions between PTH and genetic status may have been missed due to the small number of mice studied. For example, using WT + iPTH versus *Phospho1* KO + iPTH cortical BV/TV data, a calculation of the sample size for a power of 80% indicated that we would require 10 mice in each group to detect a significant difference (*p* < .05). The blunting of the anabolic response in *Phospho1* deficient mice may be related to the inability of iPTH to upregulate osteoblast transcription factors such *Runx2* and *Trps1* and drive bone formation in *Phospho1* KO mice. Alternatively, as PHOSPHO1 has a critical role in the initiation of the mineralisation process, its presence may be a prerequisite for the bone to elicit an anabolic response to iPTH. On this basis, it is tempting to speculate that the actions of iPTH on bone are at least partly dependent on the targeting of the mineralisation phase of bone formation.^51^ The presence of a poorly mineralised bone may be compounded by the inability of iPTH to elevate *Alpl* and *Smpd3* expression in the absence of *Phospho1* as both are essential for bone mineralisation.^24^, ^44^ How the absence of PHOSPHO1 influences osteoblast expression of *Smpd3 and Alpl* in response to iPTH is unclear. However, as BMP2 stimulates both *Smpd3* and *Alpl* expression, an action blunted by PTH, there may be, in a *Phospho1* deficient state, complex cross‐talk between PTH and BMP2 signalling.^27^, ^52^ is also of note that cortical porosity appeared to be exacerbated in *Phospho1* KO mice treated by iPTH‐treatment (Figure 6H). Enhanced cortical porosity is normally associated with continuous PTH exposure and this observation would suggest raised levels of resorption by iPTH treatment of *Phospho1* KO mice. Future studies should explore *Rankl*/*Opg* expression levels to establish whether the uncoupling of osteoblasts and osteoclasts is mediating some of these effects on bone mass in the *Phospho1* KO mice.

In summary, the response of *Phospho1, Alpl* and *Smpd3* expression by cultured osteoblasts to iPTH was at odds with that noted in vivo. The increased *Phospho1, Alpl, Enpp1* and *Smpd3* expression in bone from mice subjected to long‐term iPTH treatment was consistent with an anabolic PTH regimen. In the absence of PHOSPHO1, the iPTH anabolic response was attenuated, suggesting that amplified *Phospho1* expression in response to iPTH is a prerequisite for an anabolic response.

## CONFLICT OF INTEREST

The authors declare no conflict of interest.

## Data Availability

All data supporting the findings of this study are available within the article.
